# Object Positioning Algorithm Based on Multidimensional Scaling and Optimization for Synthetic Gesture Data Generation

**DOI:** 10.3390/s21175923

**Published:** 2021-09-03

**Authors:** Borja Saez-Mingorance, Antonio Escobar-Molero, Javier Mendez-Gomez, Encarnacion Castillo-Morales, Diego P. Morales-Santos

**Affiliations:** 1 Infineon Technologies AG, Am Campeon 1-15, 85579 Neubiberg, Germany; Borja.SaezMingorance@infineon.com (B.S.-M.); Antonio.Escobar@infineon.com (A.E.-M.); Javier.MendezGomez@infineon.com (J.M.-G.); 2 Department of Electronic and Computer Technology, University of Granada, Avenida de Fuente Nueva s/n, 18071 Granada, Spain; encas@ugr.es; 3 RedNodeLabs UG, 80469 Munich, Germany

**Keywords:** infrastructure positioning, object positioning, multidimensional scaling, trajectory optimization, ultrasound, synthetic data generation

## Abstract

This work studies the feasibility of a novel two-step algorithm for infrastructure and object positioning, using pairwise distances. The proposal is based on the optimization algorithms, Scaling-by-Majorizing-a-Complicated-Function and the Limited-Memory-Broyden-Fletcher-Goldfarb-Shannon. A qualitative evaluation of these algorithms is performed for 3D positioning. As the final stage, smoothing filtering techniques are applied to estimate the trajectory, from the previously obtained positions. This approach can also be used as a synthetic gesture data generator framework. This framework is independent from the hardware and can be used to simulate the estimation of trajectories from noisy distances gathered with a large range of sensors by modifying the noise properties of the initial distances. The framework is validated, using a system of ultrasound transceivers. The results show this framework to be an efficient and simple positioning and filtering approach, accurately reconstructing the real path followed by the mobile object while maintaining low latency. Furthermore, these capabilities can be exploited by using the proposed algorithms for synthetic data generation, as demonstrated in this work, where synthetic ultrasound gesture data are generated.

## 1. Introduction

Ultrasound technology is widely used for object positioning. Applications such as robot navigation [1], indoor navigation [2], human-device interface systems [3], body-tracking [4] or medical-probes tracking [5] are just a few examples of the many potential applications based on ultrasonic waves.

Positioning systems usually have a set of fixed anchor nodes that defines the infrastructure for the location. To locate the target, there are mainly two different approaches:

1. Locating an active object, one able to emit and/or receive ultrasonic signals [6,7].
2. Locating a passive object, one which just reflects the incoming ultrasonic wave emitted by the anchors [8].

Within the active-object alternative, one option is to use the anchors as receivers and the mobile node as the signal emitter. Based on the Time of Flight (ToF) or on the Received Signal Strength Indicator (RSSI), the anchors calculate their distance to the object [9]. Another alternative is using the Angle of Arrival (AoA) [10], where the position is obtained from the direction of arrival of the signal to the receiver. This work focuses on mechanisms based on ToF measurements, which are generally more robust and accurate, by relying on the predictable velocity of the ultrasonic wave in the air. If a ToF-based mechanism is used, all the anchors require an additional synchronization mechanism to have a common clock reference. Similarly, the roles can be inverted and the anchors can synchronously transmit beacons, with the mobile node as the receiver, which computes the distances locally. To achieve the time synchronization between the nodes, a combination of different technologies may be used in the same system, such as ultra wide band (UWB) and ultrasounds [11]. Another popular approach to avoid the requirement of having a tight synchronization mechanism between the anchors is to use two-way ranging mechanisms [12] in which either the mobile node or the anchors reply with another signal after a fixed amount of time and the one-to-one distances are computed based on the individual round-trip times.

There are works that use the active-object approach, based on ultrasound technology, for positioning and tracking: Chen H. et al. [9] proposed a system, where the positioning is based in a fixed receiver array performing the localization of a transmitter array attached to the hand of the user. Chen J. et al. [13] described using ultrasonic signal and radio signal together to develop a transmitting 3D pen, and the algorithm to position the pen based on a set of receiving nodes covering the writing plane.

In the passive-object alternative, just the echo, or reflected wave, is detected back by the anchors. This is typically feasible for very short-range applications, such as gesture recognition [14], in which the surface to locate is interference-free and has a reflective surface large enough to be easily recognized. The same distance measuring techniques used in the active alternative can be used with the passive alternative [15], taking into account the characteristics of the passive approach.

Ultimately, the accuracy and robustness of the system rely on the dependability of the distance measurements. It is critical to recognize the incoming signal (either reflected or actively transmitted by another node) over the ultrasonic background noise. Several methods are proposed in the literature based on different criteria (time, frequency, phase) [16], with the most popular being the cross correlation of the received and expected signal. It requires low computational power, introduces low delay and offers higher robustness against noise when detecting an echo [13,16].

This technique enables the emission of different signals (i.e., in the case where the anchors play the emitter role) to differentiate between incoming pulses, such as Direct Sequence Code-Division Multiple Access (DS-CDMA) [17,18].

Once the distances to the anchors are obtained, the location of the object can be determined, using a positioning algorithm, one based on the trilateration concept [19,20]. Knowing the position of three anchors $A_{1}\left(x_{1},y_{1},z_{1}\right)$, $A_{2}\left(x_{2},y_{2},z_{2}\right)$, $A_{3}\left(x_{3},y_{3},z_{3}\right)$ and the pairwise distances ($d_{1}$,$d_{2}$ and $d_{3}$), the coordinates of the object, O(x,y,z) can be calculated solving the following system of equations:

(1) $$\left\{\begin{array}{c} {\left(x-x_{1}\right)}^{2}+{\left(y-y_{1}\right)}^{2}+{\left(z-z_{1}\right)}^{2}=d_{1}^{2}\\ {\left(x-x_{2}\right)}^{2}+{\left(y-y_{2}\right)}^{2}+{\left(z-z_{2}\right)}^{2}=d_{2}^{2}\\ {\left(x-x_{3}\right)}^{2}+{\left(y-y_{3}\right)}^{2}+{\left(z-z_{3}\right)}^{2}=d_{3}^{2} \end{array}\right.$$

This algebraic solution corresponds to the cross points of the three spheres with center $A_{1}$, $A_{2}$ and $A_{3}$, and radii $d_{1}$, $d_{2}$ and $d_{3}$, respectively.

Three anchor nodes, and the distance from all three anchors to the object, are needed as a minimum requirement to obtain the 3D location of the object. If there are fewer than three distances to the anchor nodes (i.e., there is no direct acoustic channel between the object and one anchor), it is not possible to determine the location.

When there are more than three anchors involved in the location, we have an overdetermined system, and the method is called multilateration. Its advantage is a potentially increased robustness against inaccurate or missing distances. With *N* anchors, it is required to solve a system with *N* equations, making necessary the use of recursive algorithms to obtain an optimal solution [21]:

(2) $$\left\{\begin{array}{c} \;{\left(x-x_{1}\right)}^{2}+{\left(y-y_{1}\right)}^{2}+{\left(z-z_{1}\right)}^{2}=d_{1}^{2}\\ \;{\left(x-x_{2}\right)}^{2}+{\left(y-y_{2}\right)}^{2}+{\left(z-z_{2}\right)}^{2}=d_{2}^{2}\\ \;\;\;\;\;\;\;\;\;\;\;\;\;\;\;\;\;\;\;\;\;\;\;\;\;\;\vdots \\ {\left(x-x_{N}\right)}^{2}+{\left(y-y_{N}\right)}^{2}+{\left(z-z_{N}\right)}^{2}=d_{N}^{2} \end{array}\right.$$

To compute the coordinates of the object, and even to previously or simultaneously position the anchor infrastructure, fast and robust algorithms are required. They should be able to easily adapt to a varying number of noisy distances, and therefore are not totally reliable. Furthermore, if trajectories are to be obtained, a further processing step is useful to smooth out the path of the object and improve the accuracy of the estimated track.

In this paper, several approaches to achieve the object location and tracking are proposed using multidimensional scaling (MDS) and optimization algorithms. A qualitative evaluation of these algorithms is performed in this work. In addition, the integration of the algorithms in a synthetic data generation framework is discussed. This use case shows how the dataset creation task, i.e., ultrasound gesture dataset, could benefit from these algorithms due to the high flexibility to configure the desired output with different noise levels and gesture options. At the same time, since the desired data are configured by the user, this framework would generate simultaneously data and labels. By applying this framework, the possibility of incurring human error is reduced, as is the required time to generate synthetic labeled datasets.

MDS localization techniques have been previously researched, mostly for technologies such as Wireless Sensor Networks (WSN), Radio or 5G [22]. However, to the best of our knowledge, these techniques have not been evaluated in emerging techniques, such as ultrasound for airborne applications. Because of this, the aim of this work is the usage of this algorithm for ultrasound data for target localization.

This work is structured as follows: Section 2 presents the objectives to cover in this work. Section 3 explains the proposed new algorithms to perform both the infrastructure and target positioning. Section 3.4 explains the filter techniques studied in this work for smoothing out the trajectory, and Section 4 describes the simulation performed. Section 5 summarizes the results obtained, focusing on different parameters of each algorithm, and methods to improve the results via filtering or changing the infrastructure layout. Finally, Section 6 presents the conclusions of this work.

## 2. Envisioned System

The goal of the present work is to analyze the feasibility and performance of a synthetic data generation framework based on the researched algorithms, due to its capabilities to accurately generate numerical samples. The required input for the data generation is an initial selection of the followed path (equation or time series of the desired movement). At the same time, this framework would enable the user to generate a more varied dataset since the noise level can be controlled as well as different modifications of the initial data (including rotating, scaling and translating the samples) in the 3D axis, which can later be converted to different formats to fit the specific application, i.e., images or voxels.

This framework could ease data gathering tasks, as real sensors are not required for this process, and it can generate numerous relevant samples that emulate different scenarios/technologies based on the configuration selected by the user, such as the anchor distribution and noise levels.

This system would be beneficial for tasks, such as gesture recognition based on multiple technologies, which numerous authors are researching. Most of the studies in this field are focused on radar [15,23], Wi-Fi [24] and ultrasound sensors [9,13,25]. In this paper, the framework will be evaluated for the generation of ultrasound data for gesture recognition. This technology is selected due to the emerging techniques with ultrasound sensors, which could be implemented directly on simple microcontroller-based devices, like that proposed in [25].

**Figure 1:**
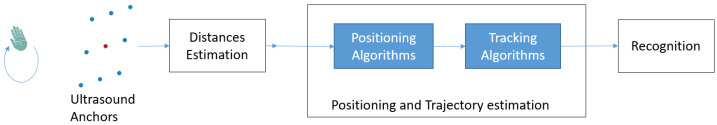
Ultrasound positioning system used as a reference for the simulations.

The system to be emulated with the proposed framework is assumed to perform the following tasks (Figure 1):

1. Distance estimation. The devices use ultrasound transceiver(s) to locally compute their distances to an object, e.g., the user’s hand, typically using ToF-based measurements. The pairwise distances between the anchors are also computed (with a lower frequency) to self-locate the anchor infrastructure.
2. Positioning algorithms. Using the pairwise distances between the anchors obtained in the previous point, the position of each anchor is computed. Then, using these positions and the distances between the user’s hand and all the anchors, the current position of the object is computed.
3. Tracking algorithms. The position of the object is periodically updated, effectively obtaining an estimation of its trajectory. This trajectory is filtered to improve its accuracy.
4. Recognition. The estimated trajectory is used as input for a gesture recognition stage, e.g., implemented with a neural network.

The current work focuses on the second and third steps, in which we transform from a temporal series of distances to the 3D trajectory of the object and the 3D positions of the anchors. It is important to say that, even when in this paper, the localization algorithms are tested with synthetic data, the proposed algorithms could also be deployed in a real scenario for target positioning.

To evaluate the applicability of the proposed algorithms, the following criteria are used:

1. The computational requirements of the positioning and tracking algorithms must be low enough to be executed in real time on low-power devices. Furthermore, they must be flexible enough to adapt to time-varying and noisy conditions, with a potentially variable number of anchors in range.
2. Analyze the accuracy of both the estimated object’s trajectory and the anchor’s position. The precision of the measured data will directly affect the results when using a classification algorithm to study the data. Because of this, it is important to ensure the high performance of the localization algorithms as well as the proposed filtering techniques. To evaluate this, noise—as typically encountered in ultrasounds systems in this case—is added to the raw distances. The positioning and tracking algorithms must provide optimal estimations and a robust behavior in the presence of noise, missing distances and outliers.

## 3. Infrastructure and Object Positioning Using Pairwise Distances

As presented in Section 1, classical trilateration techniques calculate the position of an object based on the measurement of its distance from different reference points, or anchors, which define the location infrastructure. The algorithms used for infrastructure and object positioning using pairwise distances will be described in this section, presenting the novel two-step approach proposed in this work.

### 3.1. Infrastructure Positioning Using Multidimensional Scaling

For many applications, in which the infrastructure may be portable and flexible, or a quick and seamless deployment is desired, the positions of the anchors may not be known beforehand.

A self-positioning infrastructure, in which relative coordinates of the anchors are obtained from their pairwise distances, can be achieved by using metric multidimensional scaling (mMDS) techniques. All the pairwise Euclidean distances among the anchors shape the dissimilarity matrix, which is then used to calculate the relative coordinates of the anchors by minimizing a stress function based on iterative metric-preserving techniques [26]. The scaling by majorizing a complicated function (SMACOF) algorithm is proposed for a computationally-efficient resolution of the problem [27]. Distances, or equivalently dissimilarities, are considered noisy, and some pairs may be missing. To account for different degrees of confidence in the dissimilarities, they can be weighted differently, e.g., from zero (distance is considered missing and ignored during the stress computation) to one. Due to the noisy nature of real-world distance measurements, an analytical exact solution is usually not available, and iterative techniques, such as SMACOF, are more suitable.

Different approaches based on MDS are proposed in the literature to improve the computation of the coordinates [28], such as matrix completion, in which missing distances are estimated (e.g., with Dijkstra’s shortest path algorithm) instead of being given zero weight. Another is *out-of-sample* MDS [29,30], in which the position of a subset of anchors (landmarks) can be fixed and only the remaining positions are computed. Furthermore, mixing different steps of non-metric [31] and metric MDS computations can be beneficial, particularly if the dissimilarities are not directly proportional to the Euclidean distances (e.g., they are based on RSSI measurements [32]). The initialization of the SMACOF algorithm may also impact the accuracy of the solution, and it is usual to run it with multiple random initializations and keep the solution with lower stress [33].

The result of the MDS algorithm is a cloud of points, one for every anchor. There is a set of transformations (translation, rotation and reflection) that can be applied to these points, without modifying the stress, which results in an infinite amount of equally valid solutions. The last step is to use physical constraints or general knowledge about how and where the anchors are deployed to apply these transformations and fix the coordinate system to go from relative to absolute positions [34].

### 3.2. Object Positioning Using Multidimensional Scaling

Once the coordinates of the anchors are computed, the second step is the addition of the pairwise distances of the moving objects to the dissimilarity matrix, ideally using the *out-of-sample* variation of the MDS algorithm, in order to keep the anchor positions fixed [29]. Nevertheless, MDS is computationally expensive, and while it might be the optimal solution for low-frequency infrastructure positioning, it could be too slow for high-frequency positioning of moving objects, particularly in edge computing and low power environments.

As an alternative, the position of the fixed anchors and the moving objects can be simultaneously computed. The main disadvantage of this approach is that normally the distance measurements between the fixed anchors are more reliable than the measurements between the anchors and the moving objects, e.g., distances between the anchors can be heavily averaged for noise reduction. Introducing noisier dissimilarities in the matrix affects the accuracy of the overall positioning, including that of the infrastructure, resulting in worse results than with the two-step (first MDS without mobile objects, then *out-of-sample* MDS) approach. Furthermore, the fixed infrastructure does not need to be re-positioned as fast as the objects, so it is useful to decouple both computations.

### 3.3. Object Positioning Using Optimization Algorithms

A more efficient approach for object positioning is to compute only the coordinates of the moving objects at a faster rate, using a classical optimization algorithm, based on the anchor coordinates previously obtained with MDS. In our case, we choose the Limited-Memory Broyden Fletcher Goldfarb Shanno (LM-BFGS) algorithm [35,36] with the mean squared error as the objective function to be minimized. The processing and memory requirements of LM-BFGS are low-enough to be run in real-time in low-power edge devices with a typical number of distances to the anchors (fewer than a dozen) [37].

The accuracy of this approach is comparable to the *out-of-sample* MDS one since the positions of the anchors are considered fixed during the optimization iterations, but it is faster for independently obtaining the coordinates of individual objects. Furthermore, the error is typically lower than when using the *one-step* MDS approach, in which the coordinates of the anchors and mobile objects are computed at the same time since the pairwise distances involving the mobile objects are normally noisier.

### 3.4. Trajectory Optimization Using Smoothing Techniques

The coordinates of the moving object create a (typically noisy) trajectory that benefits from proper filtering in order to provide a more accurate estimation for the final application, which could allow real-time localization or hand-gesture recognition. Different techniques are widely used for low-pass filtering and outlier detection. In our approach, we compare simple moving-average and moving-median filters [38] with a fixed window length determined heuristically. They provide optimal results, while keeping both the computational cost and low complexity.

To obtain the trajectory of a moving object, we compute its position periodically, building a discrete time-series of successive equally spaced points in time. Since additional information about the expected path is usually known, such as the maximum velocity of the object, we can exploit this to further filter the trajectory, smoothing it out, restoring missing points by interpolation, and decreasing or removing the effect of outliers in the position time-series. We explore two filtering alternatives:

1. Moving-average filter. It is a low-pass filter that provides effective noise reduction, particularly in applications where the focus is on time-response (instead of frequency-response) analysis. It smooths the signal, but the predicted trajectory may fail to respond to quick movements.
2. Moving-median filter. The median filter is a non-linear filter that replaces the values in data with the moving median of the filtered and neighboring points. It is very robust against outliers and in suppressing spiky noise, but as with the moving-average filter filtering, it may lead to an underestimation of the path, particularly in sharp corners.

## 4. Simulation Setup

**Figure 2:**
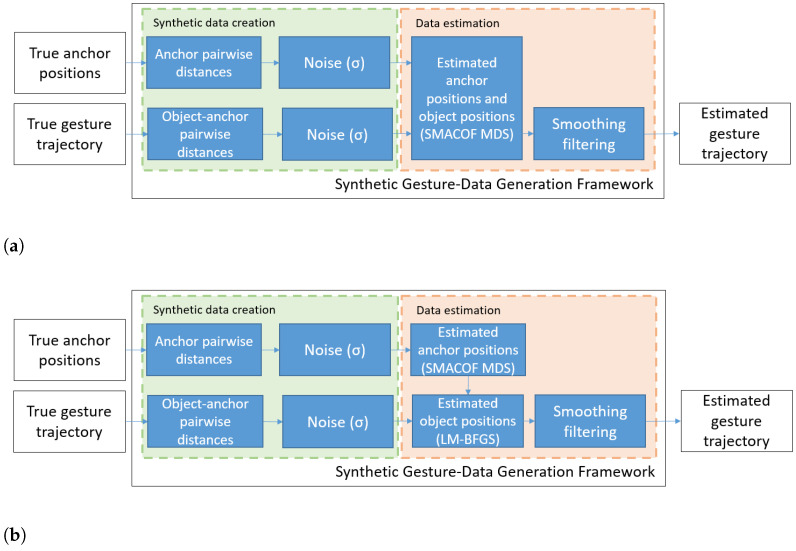
Proposed simulation framework, with (**a**) One-step approach and (**b**) Two-step approach, for the generation of synthetic gesture data.

After presenting the different alternatives for object and infrastructure positioning based on pairwise distance measurements, we estimate the performance of the proposed algorithms in terms of accuracy and execution speed. We compare the two different approaches depicted in Figure 2:

1. One-step approach (Figure 2a). The SMACOF MDS algorithm is used to simultaneously obtain the positions of the anchors and the moving objects. It is expected to be slower and less accurate if noisy dissimilarities (such as those between the moving objects) are introduced in the computation, but all the anchors and object positions are computed simultaneously.
2. Two-step approach (Figure 2b). The SMACOF MDS algorithm is used once to obtain the positions of the anchors. Then, the LM-BFGS optimization algorithm is used to compute only the coordinates of the moving object, and repeated periodically to update its position. This approach is faster, but relies on an accurate initial estimation of the anchor positions.

By using these two algorithms, it is possible to design the proposed framework for synthetic data generation. It executes the SMACOF MDS algorithm periodically to ensure that the position of the anchors is correct while locating simultaneously the target. Between these anchors check, the LM-BFGS algorithm is used due to its low latency and high accuracy when the position of the anchors is known.This approach is faster and results in a smaller error, as we will discuss in Section 5.2 and Section 5.4. The SMACOF MDS and LM-BFGS optimization computation steps can be done in a central processing node to which all the anchors report, or it can be done locally in the anchors or the mobile object, if they have access to all the distances. The particular communication scheme to disseminate the distances and the positions is out of the scope of this work. Finally, if we want to estimate a path and not only the single positions, a smoothing filter is used to compute the trajectory of the object.

Consequently, this framework can be used to generate synthetic trajectories for an arbitrary number of anchor configurations and gestures to fit multiple scenarios and applications, as shown in the synthetic data creation block in Figure 2. At the same time, data augmentation for a single gesture and anchors setup is possible by varying the random initialization seeds of the noise for the SMACOF MDS and the LM-BFGS optimization algorithms, as shown in the data estimation block in Figure 2. Consequently, this framework can efficiently generate numerous samples of the desired data to contemplate all the possible results of measurements with real devices. Furthermore, different noise models and strengths can be injected to the raw distances, emulating different disturbances and inaccuracies that the ultrasound distance gathering system can experience in a real deployment.

### 4.1. System Modeling

In this work, the framework emulates a system of nine ultrasound anchors sending a sinusoidal pulse (reference pulse). The echoes are then sampled, and the ToF is obtained with classical cross-correlation techniques, using a reference signal. Every pairwise distance is computed at 20 Hz, i.e., a new target position is computed every 50 ms. A two-step approach to compute the anchor and target positions as depicted in Figure 2.

**Figure 3:**
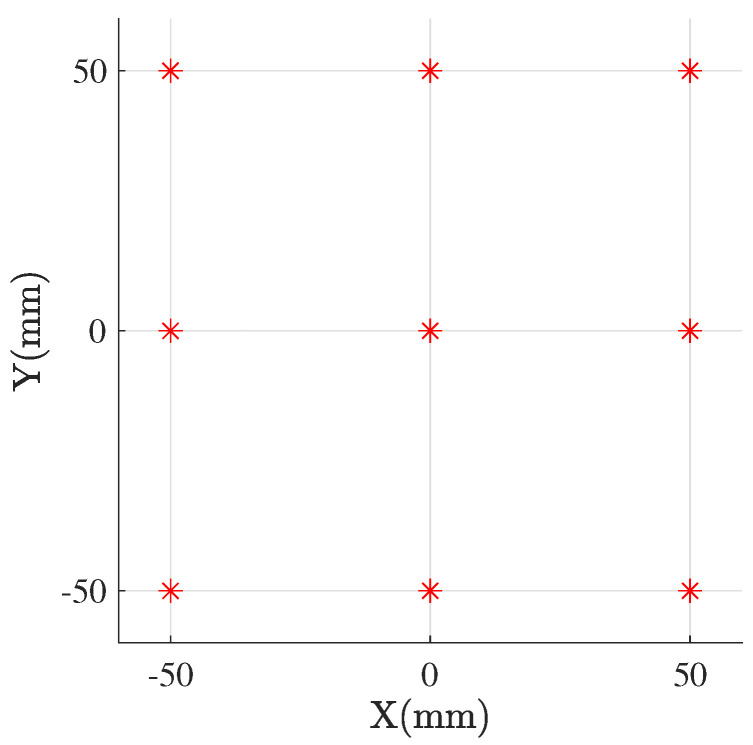
Position of the ultrasound anchors.

The infrastructure of the anchors for the proposed framework is shaped as a 2D array of nine anchors (ultrasound transceivers) located in the same surface (in the XY plane with z=0), which represents a plausible configuration for future applications. Specifically, the anchors are located as seen in Figure 3. The positioning is limited to the space in front of said surface (z>0) since the sign of the *z* coordinate cannot be defined when all the anchors are in the same plane. Furthermore, the ultrasound transceivers sensors used as an experimental support for the simulations have a detection range limited to 180 degrees in the Z-axis.

The anchor array is able to transmit and receive ultrasonic signals and locate passive objects based on ToF measurements. It has two operating modes:

1. To calculate the pairwise distances between the anchors, they actively exchange ultrasonic signals (two-way ranging).
2. To calculate the pairwise distances between the anchors and the mobile object, they actively transmit and then sense the reflected echo. Anchors can be synchronized, in which case, only one of the transceivers needs to transmit and they all can receive the echo and timestamp it based on a common clock. Otherwise, they can all transmit and sense only the echo coming from their own transmission; in such a case, time synchronization is not required.

### 4.2. Noise Modeling

The noise in the distances obtained with an ultrasound-based measurement system depends on the accuracy of the ToF samples. There are different factors that impact the performance, such as the bandwidth of the transmitted pulse and the sampling rate of the acquisition stage.

Based on our experimental measurements using the system of Figure 1, the noise, *N*, in the computed distances, d(t), can be modeled as unbiased (zero average) additive white Gaussian noise (AWGN), with a given standard deviation, σ, and a probability density function, pdf(N), given by the following:

(3) $$\begin{array}{cc} d(t) & =d_{real}\left(t\right)+N\left(t\right) \end{array}$$

(4) $$\begin{array}{cc} pdf(N) & =\frac{1}{\sigma \sqrt{2\pi }}\exp\left(-\frac{1}{2}{\left(\frac{x}{\sigma }\right)}^{2}\right) \end{array}$$

**Figure 4:**
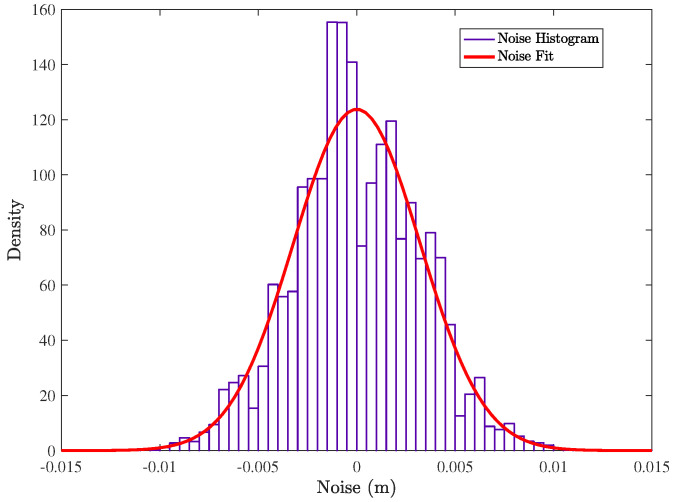
Histogram of the measured noise in experimentally-computed distances and normal distribution fit (σ=3.2 mm).

In a representative x-y-z point, P=(0cm,0cm,50cm), the measured equivalent noise in the Euclidean distance, after acquiring 50,000 samples, can be fitted with a σ=3.2 mm, as seen in Figure 4. This provides a good estimation of the scale of the expected noise in a real system, and it is used as a reference to model the noise in the simulations.

As a summary, in this section, the two-steps algorithm proposed and evaluated in this work is described, as well as the steps performed to particularize the framework for validate the use of ultrasonic system as technology for that algorithm.

## 5. Performance Results

In this section, the performance of the proposed algorithms for the object position and anchors localization is analyzed. At the same time, the utility of the proposed system for data generation tasks is discussed.

### 5.1. Infrastructure-Positioning Accuracy

First, we characterize the performance of SMACOF MDS locating the infrastructure by building the dissimilarity matrix, *D*, with the metric distances measured between the anchors, adding different realistic levels of AWGN noise, according to the experimental results obtained in Section 4.2.

Since the positions computed by SMACOF MDS are equally valid if they are translated, rotated or mirrored, we need additional constraints to fix the coordinate system. We use an approach to agree on a common absolute reference that requires one translation and three rotations:

1. We translate the points so that the first anchor, $m_{0}$, fixes the origin of the coordinates system, $P_{m_{0}}=\left(0,0,0\right)$.
2. We rotate the points around the X axis at an angle such that the second anchor, $m_{1}$, is in z=0, $P_{m_{1}}=\left(a,b,0\right)$.
3. We rotate the points around the X axis at an angle such that the second anchor, $m_{1}$, is in y=0 and c>0, $P_{m_{1}}=\left(c,0,0\right)$.
4. We rotate the points around the X axis at an angle such that the third anchor, $m_{2}$, is in z=0 and e>0, $P_{m_{2}}=\left(d,e,0\right)$.
5. In our configuration, all the anchors are in the same surface (z=0), so no additional condition is required. In general, it is required to fix the positive direction of the X-axis. If the anchors constitute a three-dimensional shape, another anchor (e.g., $m_{3}$), located in a surface different to the previous three ($m_{0}$ to $m_{2}$), is used to define the positive X direction.

**Figure 5:**
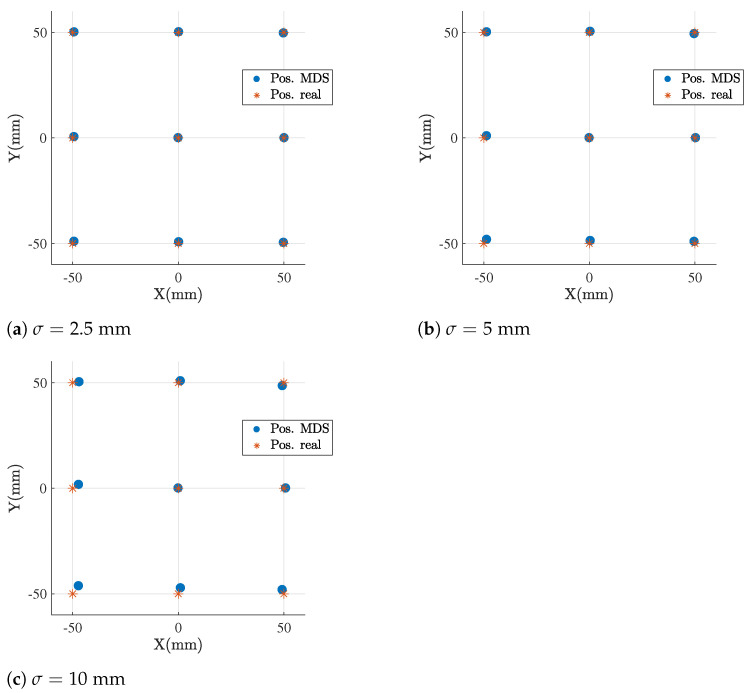
Simulated infrastructure-positioning results for different noise levels using SMACOF MDS (MDS).

Following these steps, we obtain a consistent reference system every time, removing any ambiguity in the positions. The results locating the anchor infrastructure depending on the strength, σ, of the AWGN noise in the distances are shown in Figure 5. The error on the X and Y axes between the real and calculated positions can be observed. Table 1 provides the quantitative comparison for this error in the X, Y and Z axes, and also the total displacement. We obtain an optimal accuracy, with an expected error comparable to the deviation introduced by the noise strength.

### 5.2. Object-Positioning Accuracy

**Figure 6:**
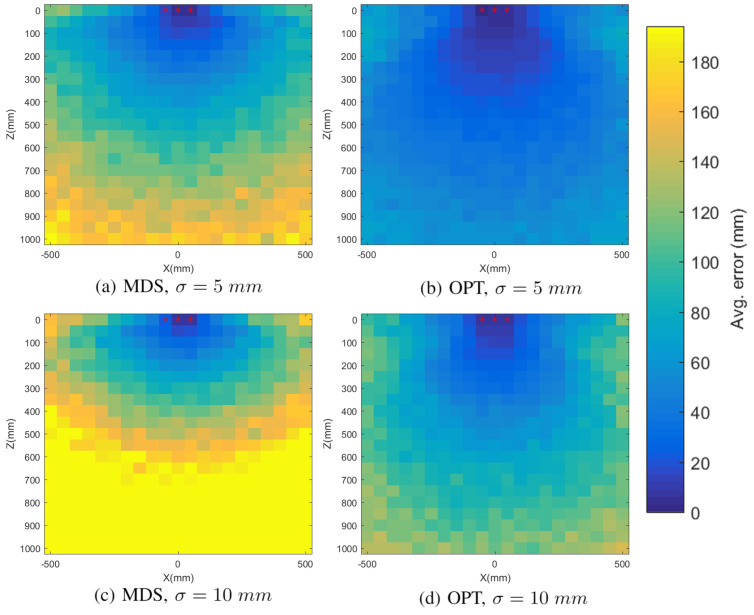
Average object-positioning error (Euclidean distance between the real and the computed point) in different regions and for different noise levels, with SMACOF MDS (MDS) and LM-BFGS optimization (OPT).

Once the anchor infrastructure is positioned, either with the MDS-based self-locating mechanisms explained in Section 5.1, or because the positions of the anchors are previously known, we can either apply LM-BFGS optimization to locate the mobile objects or reapply SMACOF MDS, adding the distance of the mobile object to the dissimilarity matrix. For the simulations, we use the anchor infrastructure of Figure 3, and repeat the positioning of 250,000 objects randomly distributed in the surface given by 0<z<1000 mm; −500 mm <x<500 mm and y=0. As shown in Figure 6 and Table 2, since the anchor infrastructure is symmetrical with respect to the z=0 surface, the averaged error in the X and Y axes is similar, while it is generally much larger for the Z axis. The chosen anchor infrastructure is flat, with no variability in the X axis. Such a configuration works well for locating in the X and Y axes, but it struggles in the Z axis. The error also increases as we move away from the anchors, being minimal in the region close to them. LM-BFGS optimization, besides being faster, has fewer errors since it only tries to optimize the position of the mobile object, without trying to minimize the error by also relocating the fixed anchors. The results further validate our simple two-step approach of using SMACOF MDS just for positioning the infrastructure and then employing LM-BFGS optimization to update the position of the mobile object.

### 5.3. Trajectory-Positioning Accuracy

As a relevant application of proposed system for synthetic data generation, we simulated the data acquisition process based on ultrasound transceivers for gesture recognition. This framework was evaluated by creating three different gestures based on some initial time series of the 3D coordinates of the trajectory.

**Figure 7:**
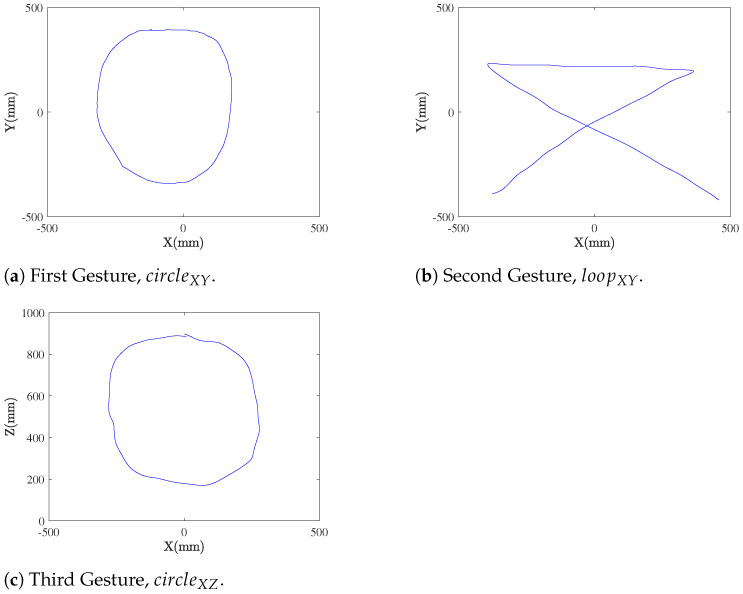
Gestures used in the simulations.

The chosen gestures are presented in Figure 7. They simulate the imperfections of real gestures. The first one (Figure 7a, $circle_{XY}$) is a circular shape in a surface with low X axis variability (100<z<120), and mostly parallel to the anchor infrastructure, to simulate a gesture in the optimal recognizing conditions. The second one (Figure 7b, $loop_{XY}$) is a gesture with sharp corners, in the same surface, to evaluate the performance of the smoothing filters in gestures with sharp edges. The third one (Figure 7c, $circle_{XZ}$) is another circular shape, this time located in a surface mostly perpendicular to the anchor infrastructure (100<y<120), to check the worst-case performance (strong *z* variability). The first gesture has 400 samples, while the second and third have 250 samples each. They are all sampled at 20 Hz.

**Figure 8:**
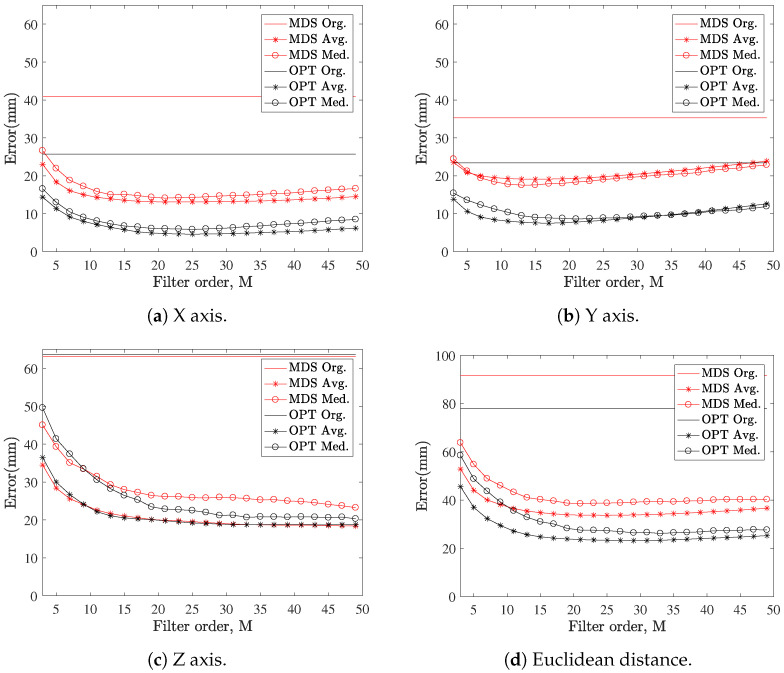
Comparison of the average trajectory error for the *cilcle_xy_* gesture, with a fixed noise level (*σ* = 10 mm), with moving-median (Med.), moving-average (Avg.) smoothing filter and without (Org.) smoothing, for SMACOF MDS (MDS) and LM-BFGS optimization (OPT) algorithms.

First of all, the frame tests different filter orders, *M*, to heuristically choose a value in which the error is properly minimized. In Figure 8, the error dependency with the filter order is shown, for both filters described (moving-median and moving-average) and for both positioning algorithms (SMACOF MDS and LM-BFGS optimization). The error without filtering is added too, as a reference. Based on those results, M=11 is selected as a good trade-off between added delay and error suppression. With the considered AWGN noise model, the moving-average filtering performs consistently better than the moving-median filter, which may change if spiky noise and outliers are introduced in the model.

**Figure 9:**
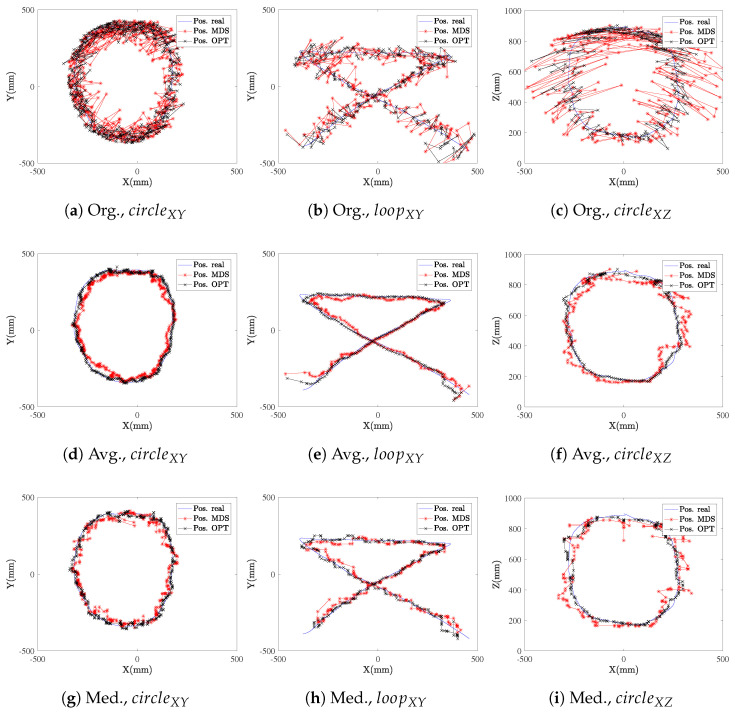
Comparison between the real and estimated trajectories for SMACOF MDS (MDS) and LM-BFGS optimization (OPT) algorithms for the three different gestures, with a fixed noise level (*σ* = 10 mm), without smoothing (sub-figures **a**–**c**), with moving-average (Avg.) smoothing (subfigures **d**–**f**) and with moving-median (Med.) smoothing (sub-figures **g**–**i**).

Without added noise (σ=0 mm), we can see in Table 3 that the smoothing filters actually deteriorate the performance since the edges are underestimated. In a realistic noisy scenario, the smoothing filter greatly reduces the error, as shown in Table 4, Table 5 and Table 6, where the average error (in each axis and in total) is compared for each algorithm, and each filtering technique previously described. As seen in Figure 9, as expected, the noisiest gesture in the surface of interest is the $circle_{XZ}$, due to it being performed in a plane normal to the anchor surface. The smoothing filtering also struggles in the sharp edges of the $loop_{XY}$ gesture, while it estimates the path with very good accuracy for the inherently smooth circular gestures.

Nevertheless, the achieved results show how the proposed algorithms are able to accurately generate the desired gesture/trajectory data with different noise levels. This would enable the framework to simulate a large range of possible scenarios and sensors. The noise level can be defined by the user to adapt the specific application and scenario that the framework is emulating.

### 5.4. Execution Time

We use the implementation of the SMACOF MDS and LM-BFGS optimization algorithms included in the Python library for machine learning *scikit-learn 0.23*, and run them in a typical Windows laptop (Intel Core i5-8350U@1.70GHz., 8GB RAM). Since absolute values depend on the processing capabilities of the particular machine, only relative time differences are evaluated. In this case, the evaluation is performed over the gesture represented in Figure 7a. As seen in Table 7, the SMACOF MDS algorithm is about ten times slower, which might be relevant for real-time applications, particularly if the position inference needs to be done directly in an embedded edge device, which typically has constrained resources. These constrains could lead to a larger difference between these algorithms, due to the computing power required to execute the SMACOF MDS algorithm. In the context of continuously positioning a slowly (relative to the positioning sampling rate) moving object, initializing the algorithm with the previous position greatly reduces (it takes half the time) the execution time of SMACOF MDS, while for LM-BFGS optimization, it barely changes.

Although SMACOF MDS is slower, it needs to be stressed that its output includes the position of all the anchors, while LM-BFGS optimization only computes the position of a single moving object. Therefore, SMACOF MDS is relevant for self-calibrating anchor infrastructures or by simultaneously locating multiple moving objects. Once the infrastructure is properly positioned during the initial set-up phase, LM-BFGS optimization can be used for quick updates of the object position.

## 6. Conclusions

This work presents a novel two-step technique to perform general infrastructure and moving-object positioning based on measured pairwise distances. In the first step, MDS is used to obtain the coordinates of the anchors, repeated with a low frequency, e.g., to correct minor and infrequent potential displacements of the anchors. We use the SMACOF variant of the mMDS family of algorithms. With the coordinates of the computed anchors computed, a fast optimization algorithm is used to obtain the unknown coordinates of the objects. This step is repeated with a high frequency. The LM-BFGS optimization algorithm is used for this step. Its performance is thoroughly analyzed with simulations, particularized to the use case of a system with ultrasound transceivers. The distribution and shape of the anchor infrastructure, the size of the region in which the positioning takes place and the strength of the noise are realistically modeled after such a system.

This two-step approach described in the work would be optimal in scenarios where the position of the anchors does not change frequently through time. Therefore, the one-step approach described in Section 4 in which all the positions are computed at the same time, is reserved for special situations, e.g., when there are no anchors (all the objects are considered mobile) or when we want to simultaneously obtain the position of several (more than a dozen) mobile objects. For the rest of the scenarios, our approach performs the localization with low computational time, making it suitable for use in real-time systems and even in constrained edge devices.

Efficient and simple filtering techniques significantly reduce the error and improve the reconstruction of the real path followed by the mobile object. This feature can be exploited when using the proposed algorithms for synthetic data generation. The current dataset creation step for applications, such as AI models, are time consuming, due to the complexity of the recording and labeling tasks, which could be reduced by using the proposed system as a synthetic data generation framework. This framework is independent from hardware and it could simulate trajectories/movement from a large range of sensors. The parameters of this framework (noise, gesture and anchors number and position) are defined by the user through the initial configurations.

The use of ultrasonic signals for target positioning is widely researched, but to the best of our knowledge, our two-step approach inspired by wireless sensor network’s positioning algorithms has not been used or described. The proposed technique enables using an arbitrary number of ultrasound transceivers, and removes the constraint of knowing the position of the anchors beforehand, while providing an optimal AWGN rejection. This could drive the adoption of ultrasound technology in the positioning field and foster the research of novel applications and electronic components based on non-audible acoustic waves.

## Figures and Tables

**Table 1:** Simulated infrastructure-positioning average error (of the nine anchors) for different noise levels with SMACOF MDS, per-axis and Euclidean distance.

		Average Positioning Error (mm)			
	Algorithm	σ = 0 mm	σ = 2.5 mm	σ = 5 mm	σ = 10 mm
X Ax.	SMACOF MDS	0	0.02	0.08	0.11
Y Ax.	SMACOF MDS	0	0.03	0.08	0.10
Z Ax.	SMACOF MDS	0	0.01	0.01	0.03
**Euc. Dis.**	SMACOF MDS	0	0.03	0.08	0.14

**Table 2:** Per-axis object-positioning error and Euclidean distance between the real and the computed point for different noise levels, with SMACOF MDS and LM-BFGS optimization, averaged for the 0<z<1000 mm; −500 mm <x<500 mm and y=0 surface.

		Average Positioning Error (mm)			
	Algorithm	σ = 0 mm	σ = 2.5 mm	σ = 5 mm	σ = 10 mm
X Ax.	SMACOF MDS	0.03	16.35	28.39	48.21
	LM-BFGS	0.01	7.64	15.21	28.69
Y Ax.	SMACOF MDS	0.03	15.67	26.98	45.54
	LM-BFGS	0.01	7.53	14.81	28.88
Z Ax.	SMACOF MDS	0.17	36.94	56.91	79.65
	LM-BFGS	0.04	23.25	42.05	67.50
**Euc. Dis.**	SMACOF MDS	0.19	47.44	75.85	114.79
	LM-BFGS	0.05	27.68	50.97	85.91

**Table 3:** Average trajectory error for the three different gestures without noise (σ=0 mm), with no smoothing (Original) and with 11th order (M=11) moving-average (Average) and moving-median (Median) smoothing filters, for SMACOF MDS and LM-BFGS optimization algorithms.

			Average Positioning Error (mm)		
			$circle_{XY}$	$loop_{XY}$	$circle_{XZ}$
**X Ax.**	SMACOF MDS	Original	0.03	0.02	0.04
		Average	0.69	2.71	1.67
		Median	0.19	1.02	0.14
	LM-BFGS	Original	0.01	0.01	0.01
		Average	0.69	2.72	1.67
		Median	0.17	1.01	0.11
Y Ax.	SMACOF MDS	Original	0.03	0.03	0.04
		Average	1.4	1.79	0.29
		Median	0.07	0.67	0.19
	LM-BFGS	Original	0.01	0.01	0.01
		Average	1.4	1.79	0.29
		Median	0.05	0.66	0.18
Z Ax.	SMACOF MDS	Original	0.08	0.09	0.02
		Average	0.34	0.36	2.17
		Median	0.22	0.28	0.38
	LM-BFGS	Original	0.02	0.02	0.01
		Average	0.34	0.34	2.17
		Median	0.19	0.26	0.37
**Euc. Dis.**	SMACOF MDS	Original	0.09	0.1	0.07
		Average	1.76	3.55	3.07
		Median	0.43	1.49	0.62
	LM-BFGS	Original	0.03	0.03	0.02
		Average	1.76	3.54	3.07
		Median	0.39	1.46	0.6

**Table 4:** Average trajectory error for the $circle_{XY}$ gesture, with no smoothing (Original) and with 11th order (M=11) moving-average (Average) and moving-median (Median) smoothing filters, for SMACOF MDS and LM-BFGS optimization algorithms.

			Average Positioning Error (mm)		
			σ = 2.5 mm	σ = 5 mm	σ = 10 mm
X Ax.	SMACOF MDS	Original	14.56	23.68	40.88
		Average	5.05	8.35	14.30
		Median	6.26	9.84	15.84
	LM-BFGS	Original	6.68	13.24	25.73
		Average	2.19	4.02	7.11
		Median	3.36	5.45	8.08
Y Ax.	SMACOF MDS	Original	13.42	21.72	35.29
		Average	4.84	9.81	19.26
		Median	6.23	9.50	17.76
	LM-BFGS	Original	6.43	12.82	23.41
		Average	2.54	4.22	8.04
		Median	3.17	5.44	10.36
Z Ax.	SMACOF MDS	Original	33.33	48.24	63.19
		Average	10.46	15.52	22.56
		Median	12.65	19.12	31.46
	LM-BFGS	Original	18.61	38.22	63.7
		Average	7.54	15.73	22.16
		Median	7.21	13.72	30.55
**Euc. Dis.**	SMACOF MDS	Original	41.93	62.72	91.61
		Average	13.72	22.02	36.5
		Median	17.01	25.79	43.27
	LM-BFGS	Original	22.28	45.34	78.02
		Average	9.02	18.02	27.12
		Median	9.68	17.39	35.56

**Table 5:** Average trajectory error for the $loop_{XY}$ gesture, with no smoothing (Original) and with 11th order (M=11) moving-average (Average) and moving-median (Median) smoothing filters, for SMACOF MDS and LM-BFGS optimization algorithms.

			Average Positioning Error (mm)		
			σ = 2.5 mm	σ = 5 mm	σ = 10 mm
X Ax.	SMACOF MDS	Original	11.44	19.33	33.62
		Average	6.28	8.23	18.59
		Median	8.95	12.01	19.29
	LM-BFGS	Original	5.30	10.18	21.5
		Average	3.26	4.55	11.02
		Median	5.10	7.88	13.77
Y Ax.	SMACOF MDS	Original	11.11	18.32	34.4
		Average	4.98	7.29	18.63
		Median	6.64	8.59	21.52
	LM-BFGS	Original	5.75	10.64	22.07
		Average	2.45	3.95	8.75
		Median	3.48	6.12	11.34
Z Ax.	SMACOF MDS	Original	29.62	42.04	62.45
		Average	11.73	13.75	37.2
		Median	15.14	16.37	42.33
	LM-BFGS	Original	18.9	32.85	53.58
		Average	6.71	13.07	17.92
		Median	8.80	13.14	24.65
**Euc. Dis.**	SMACOF MDS	Original	36.09	53.85	85.89
		Average	15.48	19.33	48.43
		Median	20.77	24.9	55.18
	LM-BFGS	Original	21.92	38.82	66.6
		Average	8.96	15.65	25.77
		Median	12.19	18.99	33.85

**Table 6:** Average trajectory error for the $circle_{XZ}$ gesture, with no smoothing (Original) and with 11th order (M=11) moving-average (Average) and moving-median (Median) smoothing filters, for SMACOF MDS and LM-BFGS optimization algorithms.

			Average Positioning Error (mm)		
			σ = 2.5 mm	σ = 5 mm	σ = 10 mm
X Ax.	SMACOF MDS	Original	32.46	65.27	1.5
		Average	8.98	19.12	32.04
		Median	12.25	25.04	35.4
	LM-BFGS	Original	11.74	25.61	47.97
		Average	3.97	7.80	16.2
		Median	5.31	9.45	17.85
Y Ax.	SMACOF MDS	Original	34.46	63.76	88.26
		Average	12.16	19.25	33.57
		Median	13.76	23.6	34.62
	LM-BFGS	Original	11.01	23.14	49.88
		Average	3.94	8.76	15.84
		Median	4.84	11.55	22.7
Z Ax.	SMACOF MDS	Original	12.85	26.46	47.18
		Average	5.59	14.14	33.14
		Median	7.36	12.96	32.24
	LM-BFGS	Original	4.71	9.93	19.13
		Average	2.61	4.07	9.21
		Median	3.91	5.80	9.80
**Euc. Dis.**	SMACOF MDS	Original	53.97	105.00	158.170
		Average	18.53	33.72	62.72
		Median	23.26	41.27	66.34
	LM-BFGS	Original	18.62	40.13	80.59
		Average	7.09	14.33	27.79
		Median	9.6	18.47	34.19

**Table 7:** Average computation time of individual positions after 1000 executions of the gesture of Figure 7a using SMACOF MDS and LM-BFGS optimization algorithms.

		Time (ms)
SMACOF MDS	Random initialization	162
	Previous-point initialization	77
LM-BFGS	Random initialization	16
	Previous-point initialization	15

## Data Availability

Not applicable.
